# Crystal structure of 3-bromo-4-di­methyl­amino-1-methyl-1,2,4-triazol-5(4*H*)-one

**DOI:** 10.1107/S205698901402636X

**Published:** 2015-01-01

**Authors:** Gerhard Laus, Thomas Gelbrich, Klaus Wurst, Herwig Schottenberger

**Affiliations:** a Faculty of Chemistry and Pharmacy, University of Innsbruck, 6020 Innsbruck, Austria

**Keywords:** crystal structure, 1,2,4-triazol-5(4*H*)-one, Br⋯O=C inter­actions, halogen inter­actions

## Abstract

The title compound, C_5_H_9_BrN_4_O, was obtained as a minor by-product in the synthesis of 4-di­methyl­amino-1-methyl-1,2,4-triazolin-5-one. Except for the methyl groups of the 4-dimethylamino moiety, all the non-H atoms lie on a crystallographic mirror plane." In the crystal, the mol­ecules are linked by C—Br⋯O=C inter­actions [Br⋯O = 2.877 (2) Å, C—Br⋯O = 174.6 (1)°] into infinite chains in the *c-*axis direction.

## Related literature   

For synthesis of related 4-amino-1-methyl-1,2,4-triazolin-5-ones, see: Kröger *et al.* (1965 ▸). For related structures with Br⋯O=C inter­actions, see: 5-bromo­pyrimidin-2-one (Yathirajan *et al.*, 2007 ▸); 3,5-di­bromo­pyran-2-one (Reus *et al.*, 2012 ▸); *N*-bromo­saccharin (Dolenc & Modec, 2009 ▸); *N*-bromo­succinimide (Jabay *et al.*, 1977 ▸); dibromantin (Kruszynski, 2007 ▸). For the theory of halogen inter­actions, see: Awwadi *et al.* (2006 ▸). For details of the synthesis, see: Schwärzler *et al.* (2009 ▸).
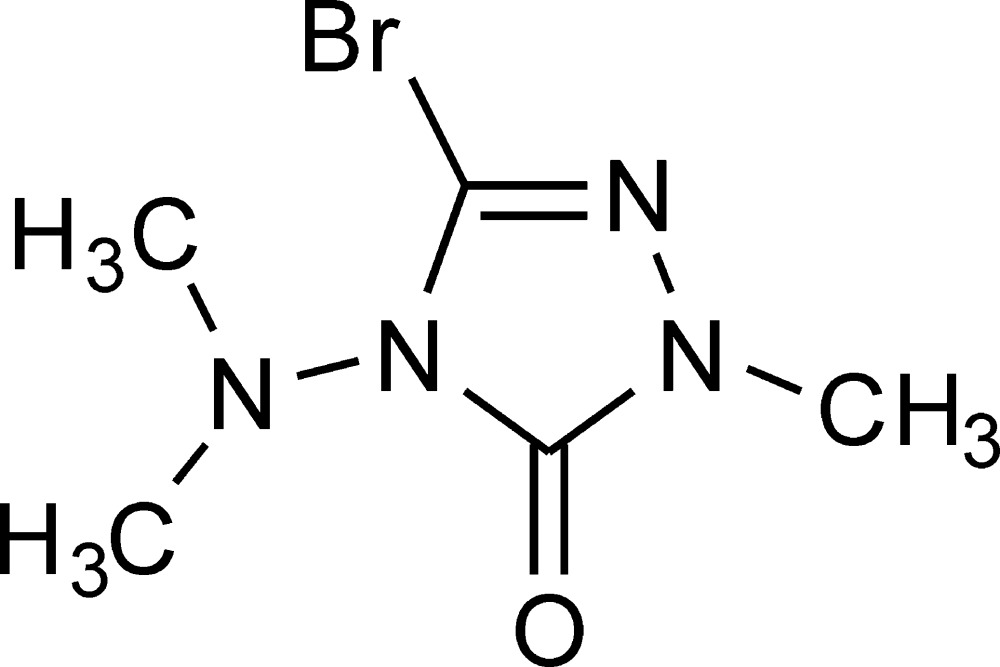


## Experimental   

### Crystal data  

C_5_H_9_BrN_4_O
*M* *_r_* = 221.07
Monoclinic, 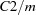

*a* = 15.1993 (6) Å
*b* = 6.9377 (4) Å
*c* = 7.8771 (7) Å
β = 93.869 (3)°
*V* = 828.73 (9) Å^3^
*Z* = 4
Mo *K*α radiation
μ = 4.91 mm^−1^
*T* = 233 K
0.09 × 0.08 × 0.07 mm

### Data collection  

Nonius KappaCCD diffractometer
2310 measured reflections
806 independent reflections
734 reflections with *I* > 2σ(*I*)
*R*_int_ = 0.034

### Refinement  

*R*[*F* ^2^ > 2σ(*F* ^2^)] = 0.028
*wR*(*F* ^2^) = 0.065
*S* = 1.07
806 reflections
75 parameters
6 restraints
H atoms treated by a mixture of independent and constrained refinement
Δρ_max_ = 0.50 e Å^−3^
Δρ_min_ = −0.44 e Å^−3^

### 

Data collection: *DENZO* (Otwinowski & Minor, 1997 ▸) and *COLLECT* (Hooft, 1998 ▸); cell refinement: *DENZO* and *COLLECT*; data reduction: *DENZO* and *COLLECT*; program(s) used to solve structure: *SHELXS97* (Sheldrick, 2008 ▸); program(s) used to refine structure: *SHELXL2014* (Sheldrick, 2008 ▸); molecular graphics: *Mercury* (Macrae *et al.*, 2006 ▸) and *ORTEP-3 for Windows* (Farrugia, 2012 ▸); software used to prepare material for publication: *publCIF* (Westrip, 2010 ▸).

## Supplementary Material

Crystal structure: contains datablock(s) I. DOI: 10.1107/S205698901402636X/fj2686sup1.cif

Structure factors: contains datablock(s) I. DOI: 10.1107/S205698901402636X/fj2686Isup2.hkl

Supporting information file. DOI: 10.1107/S205698901402636X/fj2686Isup3.mol

**Fig. 1.**
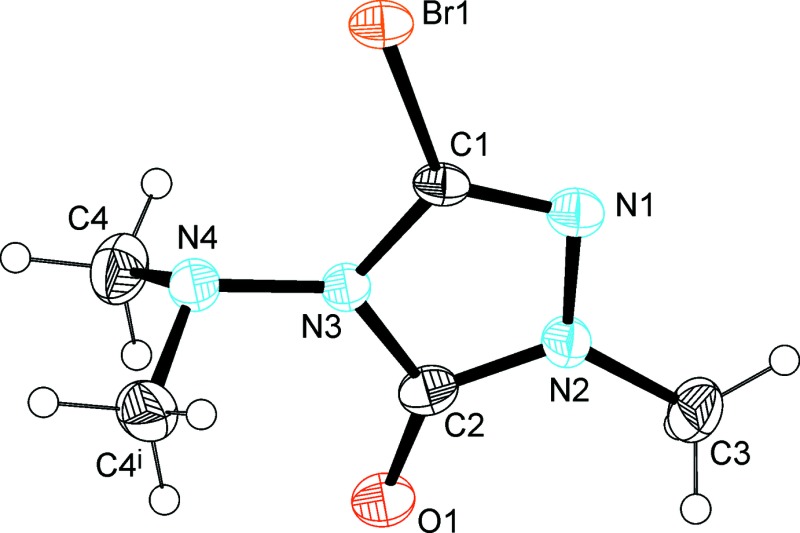
The molecular structure of the title compound, with atom labels and 50% probability displacement ellipsoids for non-H atoms. One component of the disordered C3 methyl group has been omitted for clarity. Symmetry code (i): x, -y, z.

**Fig. 2.**
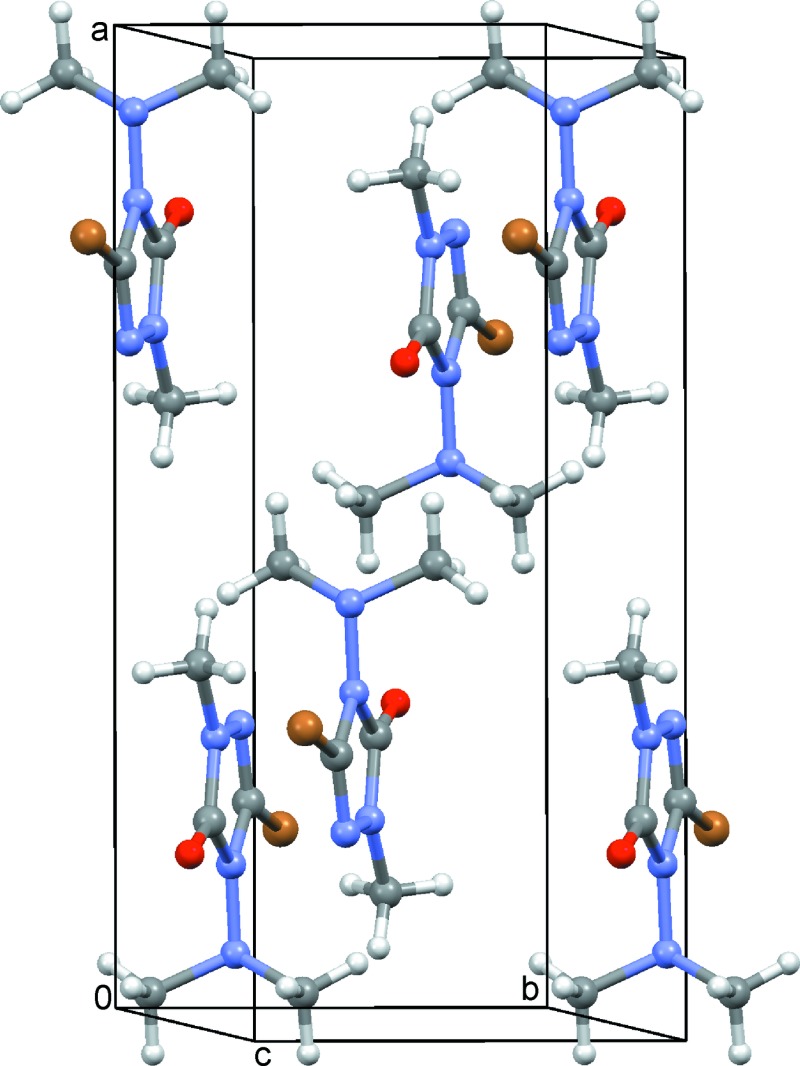
Arrangement of the triazole rings parallel to the ac plane. One component of the disordered C3 methyl group has been omitted for clarity.

**Fig. 3.**
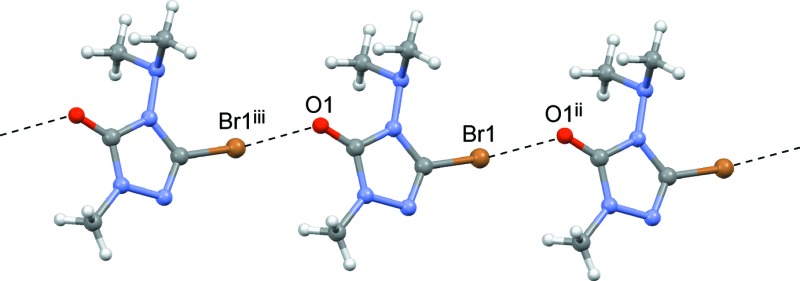
Infinite chains of molecules linked by Br···O interactions. One component of the disordered C3 methyl group has been omitted for clarity. Symmetry code (ii): x, y, 1 + z; (iii): x, y, -1 + z.

Supporting information file. DOI: 10.1107/S205698901402636X/fj2686Isup4.cml

x y z . DOI: 10.1107/S205698901402636X/fj2686fig1.tif
The mol­ecular structure of the title compound, with atom labels and 50% probability displacement ellipsoids for non-H atoms. One component of the disordered C3 methyl group has been omitted for clarity. Symmetry code (i): *x*, −*y*, *z*.

ac . DOI: 10.1107/S205698901402636X/fj2686fig2.tif
Arrangement of the triazole rings parallel to the *ac* plane. One component of the disordered C3 methyl group has been omitted for clarity.

x y z x y z . DOI: 10.1107/S205698901402636X/fj2686fig3.tif
Infinite chains of mol­ecules linked by Br⋯O inter­actions. One component of the disordered C3 methyl group has been omitted for clarity. Symmetry code (ii): *x*, *y*, 1 + *z*; (iii): *x*, *y*, −1 + *z*.

CCDC reference: 1036852

Additional supporting information:  crystallographic information; 3D view; checkCIF report

## supplementary crystallographic information

### S1. Comment

Triazolinones are of relevance due to their wide range of pesticidal activities. The molecular structure of 3-bromo-4-(dimethylamino)-1-methyl-1,2,4-triazolin-5-one is shown in Figure 1. The triazole rings are located in the crystallographic mirror plane (Figure 2), whereas the C4 methyl groups are situated out of this plane. The molecules are linked by short intermolecular C—Br···O=C contacts into infinite chains in the direction of the *c* axis (Figure 3). The Br···O distance of 2.877 (2) Å is significantly shorter than the sum of van der Waals radii. Theoretical calculations predicted negative ring and positive end cap domains of halogen atoms due to their polarizability (Awwadi *et al.*, 2006). The almost linear C—Br···O angle of 174.6 (1)° indicates an interaction involving the positive end cap of the Br atom. Thus, the Br atom acts as an electron-acceptor (X-bond donor) in this case.

### S2. Experimental

The title compound was obtained as a minor by-product in the synthesis of 4-(dimethylamino)-1-methyl-1,2,4-triazolin-5-one by hydrolysis of 5-bromo-4-(dimethylamino)-1-methyl-1,2,4-triazolium hexafluorophosphate (Schwärzler *et al.*, 2009) in MeOH/H_2_O. It is assumed that the 5-bromo compound was contaminated with a trace of the corresponding 3,5-dibromo compound which resulted in the formation of the present 3-bromo-1,2,4-triazolin-5-one.

### S3. Refinement

The H atoms were identified in a difference map and those of the C4 methyl group were idealized and included as rigid groups, allowed to rotate but not tip (C—H = 0.97 Å). The C3 methyl group was found to be disordered over two orientations related by mirror symmetry. Its H positions were refined with restrained C—H and H···H distances of 0.97 (1) Å and 1.58 (2) Å, respectively. The *U*_iso_ parameters of all H atoms were set to 1.5 *U*_eq_(C) of the parent carbon atom.

### Crystal data

**Table:**

C_5_H_9_BrN_4_O	*F*(000) = 440
*M**_r_* = 221.07	*D*_x_ = 1.772 Mg m^−^^3^
Monoclinic, *C*2/*m*	Mo *K*α radiation, λ = 0.71073 Å
*a* = 15.1993 (6) Å	Cell parameters from 3066 reflections
*b* = 6.9377 (4) Å	θ = 1.0–25.0°
*c* = 7.8771 (7) Å	µ = 4.91 mm^−^^1^
β = 93.869 (3)°	*T* = 233 K
*V* = 828.73 (9) Å^3^	Prism, colorless
*Z* = 4	0.09 × 0.08 × 0.07 mm

### Data collection

**Table:**

Nonius KappaCCD diffractometer	734 reflections with *I* > 2σ(*I*)
Radiation source: fine-focus sealed tube	*R*_int_ = 0.034
Graphite monochromator	θ_max_ = 25.1°, θ_min_ = 2.6°
phi and ω scans	*h* = −13→18
2310 measured reflections	*k* = −8→8
806 independent reflections	*l* = −9→8

### Refinement

**Table:**

Refinement on *F*^2^	6 restraints
Least-squares matrix: full	Hydrogen site location: difference Fourier map
*R*[*F*^2^ > 2σ(*F*^2^)] = 0.028	H atoms treated by a mixture of independent and constrained refinement
*wR*(*F*^2^) = 0.065	*w* = 1/[σ^2^(*F*_o_^2^) + (0.033*P*)^2^ + 0.5344*P*] where *P* = (*F*_o_^2^ + 2*F*_c_^2^)/3
*S* = 1.07	(Δ/σ)_max_ < 0.001
806 reflections	Δρ_max_ = 0.50 e Å^−^^3^
75 parameters	Δρ_min_ = −0.44 e Å^−^^3^

### Special details

**Table:**

Geometry. All e.s.d.'s (except the e.s.d. in the dihedral angle between two l.s. planes) are estimated using the full covariance matrix. The cell e.s.d.'s are taken into account individually in the estimation of e.s.d.'s in distances, angles and torsion angles; correlations between e.s.d.'s in cell parameters are only used when they are defined by crystal symmetry. An approximate (isotropic) treatment of cell e.s.d.'s is used for estimating e.s.d.'s involving l.s. planes.

### Fractional atomic coordinates and isotropic or equivalent isotropic displacement parameters (Å^2^)

**Table:**

	*x*	*y*	*z*	*U*_iso_*/*U*_eq_	Occ. (<1)
Br1	0.22218 (3)	0.0000	0.18788 (4)	0.03459 (19)	
O1	0.17449 (19)	0.0000	−0.4649 (3)	0.0426 (8)	
N1	0.3186 (2)	0.0000	−0.1029 (4)	0.0319 (8)	
N2	0.2997 (2)	0.0000	−0.2784 (4)	0.0302 (8)	
N3	0.1742 (2)	0.0000	−0.1669 (4)	0.0282 (7)	
N4	0.0841 (2)	0.0000	−0.1382 (4)	0.0329 (8)	
C1	0.2418 (3)	0.0000	−0.0415 (4)	0.0269 (9)	
C2	0.2125 (3)	0.0000	−0.3221 (4)	0.0322 (10)	
C3	0.3694 (3)	0.0000	−0.3938 (6)	0.0445 (11)	
H3A	0.352 (3)	−0.069 (5)	−0.497 (4)	0.067*	0.5
H3B	0.4248 (19)	−0.047 (6)	−0.343 (6)	0.067*	0.5
H3C	0.375 (3)	0.136 (2)	−0.420 (6)	0.067*	0.5
C4	0.04175 (19)	0.1766 (5)	−0.2044 (4)	0.0478 (8)	
H4A	0.0726	0.2877	−0.1550	0.072*	
H4B	−0.0192	0.1791	−0.1750	0.072*	
H4C	0.0438	0.1801	−0.3272	0.072*	

### Atomic displacement parameters (Å^2^)

**Table:**

	*U*^11^	*U*^22^	*U*^33^	*U*^12^	*U*^13^	*U*^23^
Br1	0.0454 (3)	0.0368 (3)	0.0211 (3)	0.000	−0.00047 (17)	0.000
O1	0.0429 (17)	0.067 (2)	0.0181 (14)	0.000	0.0024 (12)	0.000
N1	0.037 (2)	0.0330 (19)	0.0255 (17)	0.000	−0.0004 (14)	0.000
N2	0.0279 (19)	0.0367 (19)	0.0261 (17)	0.000	0.0020 (13)	0.000
N3	0.0257 (17)	0.0392 (19)	0.0197 (16)	0.000	0.0019 (12)	0.000
N4	0.0290 (18)	0.044 (2)	0.0252 (17)	0.000	0.0013 (13)	0.000
C1	0.035 (2)	0.027 (2)	0.019 (2)	0.000	−0.0026 (16)	0.000
C2	0.040 (3)	0.032 (2)	0.025 (2)	0.000	0.0051 (18)	0.000
C3	0.036 (3)	0.063 (3)	0.036 (2)	0.000	0.0124 (19)	0.000
C4	0.0402 (19)	0.059 (2)	0.0445 (18)	0.0125 (15)	0.0051 (14)	0.0066 (17)

### Geometric parameters (Å, º)

**Table:**

Br1—C1	1.851 (4)	N4—C4	1.464 (4)
O1—C2	1.230 (4)	N4—C4^i^	1.464 (4)
N1—C1	1.292 (5)	C3—H3A	0.967 (10)
N1—N2	1.392 (5)	C3—H3B	0.965 (10)
N2—C2	1.346 (5)	C3—H3C	0.969 (10)
N2—C3	1.442 (5)	C4—H4A	0.9700
N3—C1	1.377 (4)	C4—H4B	0.9700
N3—C2	1.389 (5)	C4—H4C	0.9700
N3—N4	1.403 (4)		
			
Br1···O1^ii^	2.876 (3)		
			
C1—N1—N2	103.9 (3)	O1—C2—N3	127.3 (4)
C2—N2—N1	112.8 (3)	N2—C2—N3	103.8 (3)
C2—N2—C3	126.2 (3)	N2—C3—H3A	111 (4)
N1—N2—C3	121.0 (3)	N2—C3—H3B	113 (3)
C1—N3—C2	107.1 (3)	H3A—C3—H3B	111 (2)
C1—N3—N4	125.0 (3)	N2—C3—H3C	102 (4)
C2—N3—N4	127.9 (3)	H3A—C3—H3C	109 (2)
N3—N4—C4	110.6 (2)	H3B—C3—H3C	110 (2)
N3—N4—C4^i^	110.6 (2)	N4—C4—H4A	109.5
C4—N4—C4^i^	113.7 (3)	N4—C4—H4B	109.5
N1—C1—N3	112.4 (3)	H4A—C4—H4B	109.5
N1—C1—Br1	125.0 (3)	N4—C4—H4C	109.5
N3—C1—Br1	122.6 (3)	H4A—C4—H4C	109.5
O1—C2—N2	128.9 (4)	H4B—C4—H4C	109.5

Symmetry codes: (i) *x*, −*y*, *z*; (ii) *x*, *y*, *z*+1.
