# Supplementary figures and images for: Crystal structure of 3-bromo-4-di­methyl­amino-1-methyl-1,2,4-triazol-5(4H)-one
Source: Acta Crystallogr E Crystallogr Commun. 2015 Jan 1;71(Pt 1):o23. doi: 10.1107/S205698901402636X (PMC4331846; doi:10.1107/S205698901402636X)

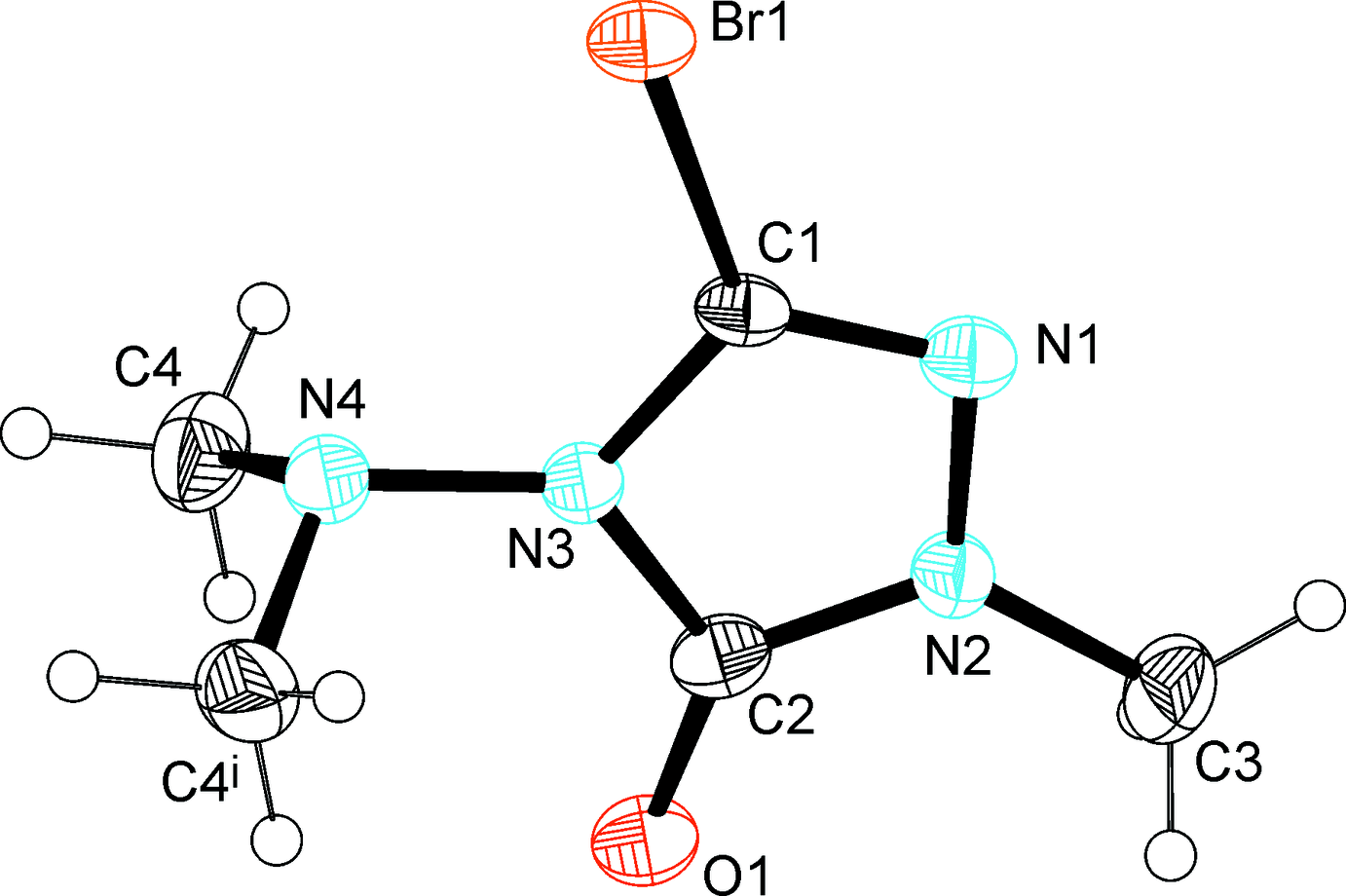

Supplement: Supplementary file 5 [file e-71-00o23-fig1.tif]

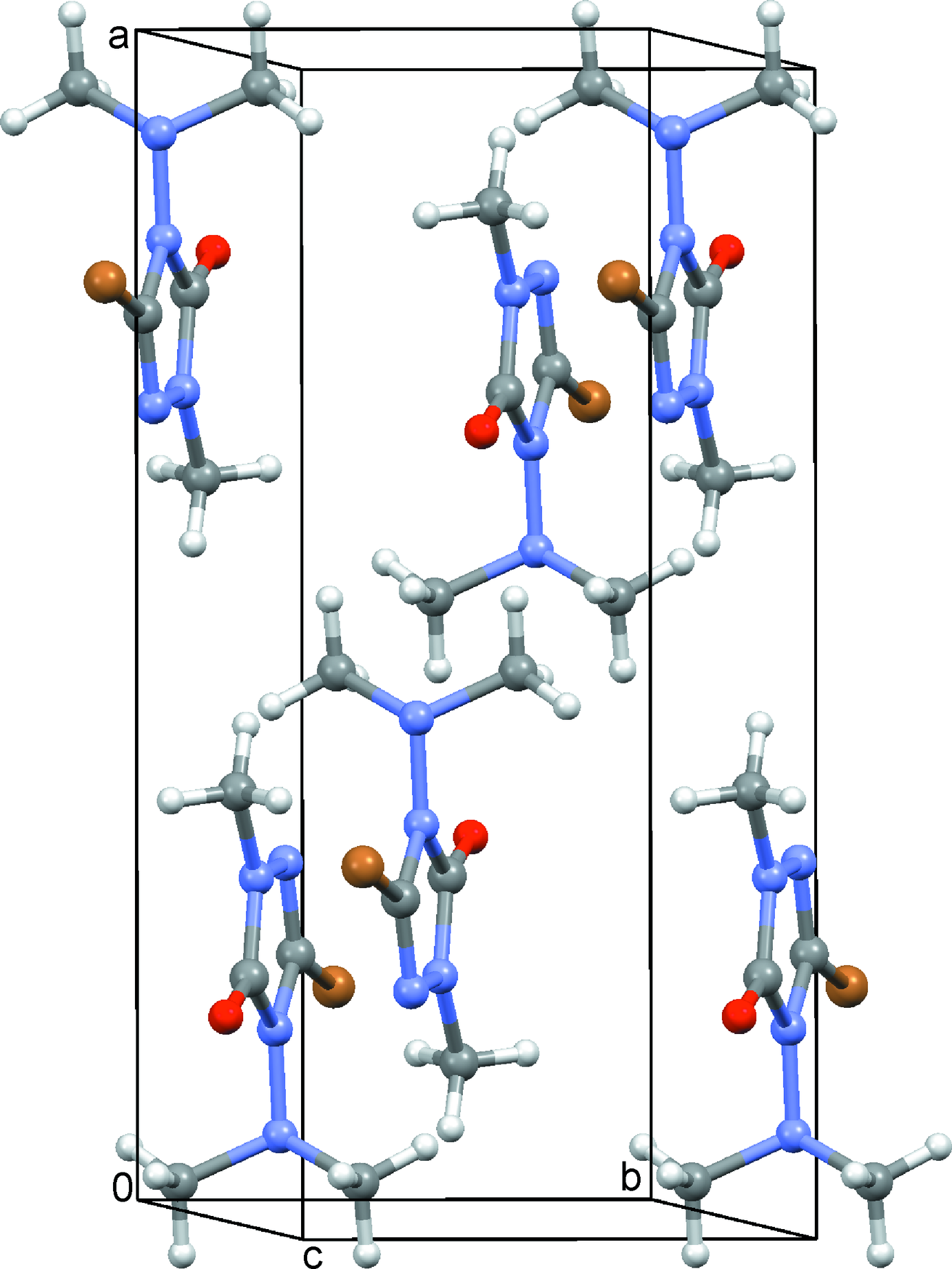

Supplement: Supplementary file 6 [file e-71-00o23-fig2.tif]

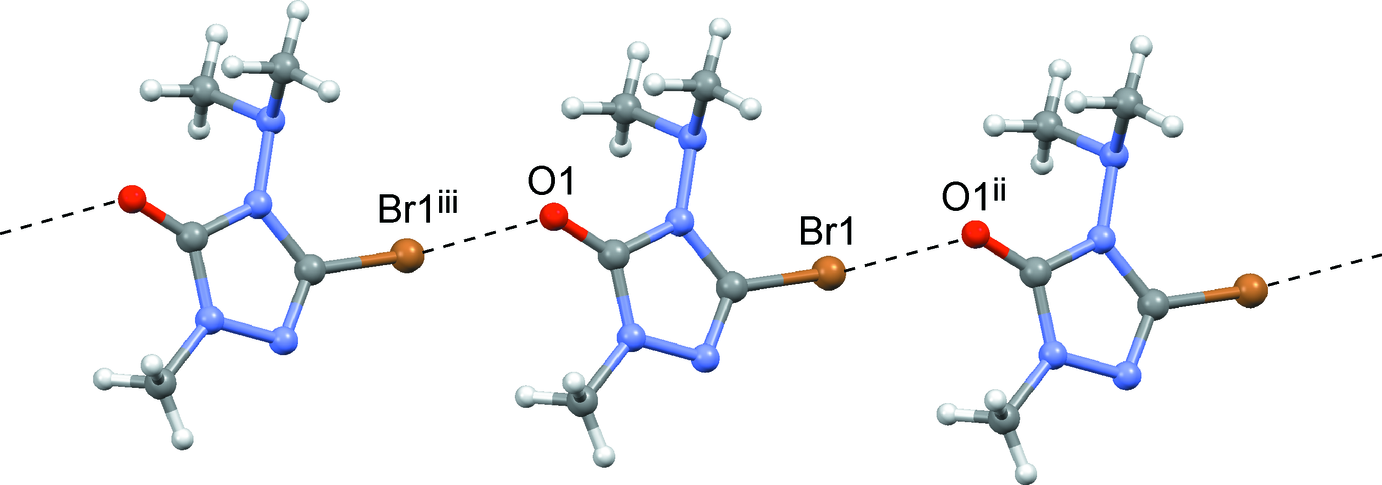

Supplement: Supplementary file 7 [file e-71-00o23-fig3.tif]
